# Social support and Quality of Life: a cross-sectional study on survivors eight months after the 2008 Wenchuan earthquake

**DOI:** 10.1186/1471-2458-10-573

**Published:** 2010-09-24

**Authors:** Xiong Ke, Chaojie Liu, Ningxiu Li

**Affiliations:** 1 West China School of Public Health, Sichuan University, Sichuan 610041, China; 2 School of Public Health, La Trobe University, Bundoora, VIC 3086, Australia

## Abstract

**Background:**

The 2008 Wenchuan earthquake resulted in extensive loss of life and physical and psychological injuries for survivors. This research examines the relationship between social support and health-related quality of life for the earthquake survivors.

**Methods:**

A multistage cluster sampling strategy was employed to select participants from 11 shelters in nine counties exposed to different degrees of earthquake damage, for a questionnaire survey. The participants were asked to complete the Short Form 36 and the Social Support Rating Scale eight months after the earthquake struck. A total of 1617 participants returned the questionnaires. The quality of life of the survivors (in the four weeks preceding the survey) was compared with that of the general population in the region. Multivariate logistic regression analysis and canonical correlation analysis were performed to determine the association between social support and quality of life.

**Results:**

The earthquake survivors reported poorer quality of life than the general population, with an average of 4.8% to 19.62% reduction in scores of the SF-36 (p < 0.001). The multivariate logistic regression analysis showed that those with stronger social support were more likely to have better quality of life. The canonical correlation analysis found that there was a discrepancy between actual social support received and perceived social support available, and the magnitude of this discrepancy was inversely related to perceived general health (rs = 0.467), and positively related to mental health (rs = 0.395).

**Conclusion:**

Social support is associated with quality of life in the survivors of the earthquake. More attention needs to be paid to increasing social support for those with poorer mental health.

## Background

An earthquake, of magnitude 8.0, struck Wenchuan in South West China on 12 May 2008. The earthquake resulted in 69,227 deaths, with another 17,923 missing and an additional 374,643 injured [1]. Earthquakes can have serious psychological impacts for survivors as well as physical injuries and the exacerbation of existing physical conditions [2].

Previous research has examined the psychological consequences of earthquakes on survivors [2-7]. However, the health hazards of earthquakes go beyond psychological consequences. Studies have also suggested that earthquakes can impair the health related quality of life (HRQoL) of the survivors [2,8,9]. Earthquake survivors are commonly forced to live in makeshift circumstances with associated stresses but appear to have poorer physical capacity and psychological wellbeing than before the earthquake [2]. The impact of an earthquake on the HRQoL of survivors could last for as long as six years after earthquake [8].

The underlying mechanisms for such long-lasting health impacts are not fully understood. Previous studies have attempted to identify risk factors associated with the poor HRQoL of survivors. Older age, female gender, financial problems, physical illness, impaired memory and decline of social activities have been identified as predictors of worsened HRQoL [7,10,11]. Damage to the properties and livelihoods are also believed to be negatively associated with HRQoL of the survivors as they have a direct impact on the capability of the survivors to cope with financial losses [2]. The connection between earthquake-induced distress and HRQoL has been the focus of health researchers in recent years. Poor quality of life is believed to be associated with mental distress and behavioral change in the aftermath of an earthquake [2,8,11]. A negative correlation has also been found between HRQoL and psychiatric impairment in the aftermath of the earthquake. The greater the severity of the psychiatric impairment, the lower the scores are for quality of life [10]. A longitudinal study of earthquake-related post traumatic stress disorder (PTSD) in a randomly selected sample in north China found that the survivors who suffered from PTSD had worse quality of life than those without PTSD [12]. Another study undertaken six months and three years after the earthquake confirmed that the variation of HRQoL of earthquake survivors is closely associated with the changes in the PTSD symptoms among the survivors [7].

As a multi-dimensional construct measuring perceived physical, mental and social functioning [13], HRQoL can be influenced by a wide range of factors, including demographic characteristics [11,14], social support [11,15], sense of coherence [16,17], people's expectations and the way the questionnaires are administered [18]. After an earthquake, it is a common practice to provide external resources including social support to help people cope with the consequences of the quake. It is generally believed that social support can buffer the negative impact of stressful events on people's wellbeing [19]. Some researchers believe that social support not only improves HRQoL directly, but it also exerts an indirect positive influence on HRQoL through facilitating post-disaster recovery among victims [20]. Despite good intentions, however, social support is not always beneficial [21]. Inadequate social support might bring no or even negative impact on people's wellbeing. Research has demonstrated that the appropriateness of social support depends on the cultural context, the life event, the characteristics of the individual and the relationship between the provider and recipient [21-23]. A study in the USA found that while Asians are more likely to benefit from implicit social support (social networking), Caucasians are more likely to benefit from explicit social support (event-specific advice) [24].

In an emergency, there is often an outpouring of social support from various external and internal sources, sometimes without careful examination of the appropriateness of such support [25]. To our knowledge, there is a paucity of research examining the relationship between social support and HRQoL in earthquake victims. This study is the first of its kind to examine the relationships between multiple dimensions of social support and HRQoL among survivors of the 2008 Wenchuan earthquake. Such research is important for policy makers to understand the consequences of earthquakes on the HRQoL of survivors and to develop appropriate strategies of social support.

This study used a cross-sectional design. We start this report of our results with a description of HRQoL and social support of the survivors of the Wenchuan earthquake. We then report on the association between social support and HRQoL among the earthquake survivors which was examined through multivariate logistic regression analysis and canonical correlation analysis.

## Methods

This research was approved by the Ethics Committee of Sichuan University.

### Study subjects

A multistage stratified and cluster sampling strategy was employed to select participants from zones within the area proclaimed by the State Council as an earthquake-hit region. The earthquake-hit region was classified into three zones: Zone One referred to the region situated in a fracture zone of the earthquake, with devastating casualties and collapse of many buildings; Zone Two referred to the region adjoining Zone One, but with fewer collapsed buildings and casualties than in Zone One; Zone Three referred to other affected areas. Three counties were randomly selected from each of the classified zones. Then, from these nine counties, a systematic sampling approach was adopted to randomly select 11 shelters, three in Zone One, six in Zone Two, and two in Zone Three. A total of 2069 people lived in the selected shelters. Residents in the selected shelters who were 16 years or older were invited to participate in the survey. A total of 1741 residents met the eligibility criteria. From 15 January to 2 February 2009, 1617 participants completed the survey, a response rate of 92.9%.

The respondents had a mean age of 32.28 years, which was younger than the national average. More men than women completed the survey. More than 40% of respondents were full time students. The sample reflected the distribution of populations in the shelters at the time of the survey, not necessarily the earthquake survivors at large (Table 1).

**Table 1:** Socio-demographic characteristics of the 1617 respondents in the survey of survivors eight months after the 2008 Wenchuan earthquake

Socio-demographic Characteristics	*n*	*%*	Average in Shelters (%)	National Average*(%)
**Age**				
16 ~	781	48.3	45.4	14.6
25 ~	184	11.4	11.9	13.5
35 ~	299	18.5	19.4	18.3
45 ~	181	11.2	11.8	15.0
55 ~	101	6.2	6.8	11.1
65 ~	71	4.4	4.7	9.5
**Gender**				
Male	864	53.4	51.6	50.8
Female	753	46.6	48.4	49.2
**Marital Status**				
Married	768	47.5	50.2	
Single	797	49.3	46.6	
Other (widowed or divorced)	52	3.2	3.2	
**Years of Education**				
0 ~ 9	723	44.7	42.9	80.0
10 ~ 12	438	27.1	28.7	13.8
Tertiary	456	28.2	28.4	6.2
**Job**				
Employed	798	49.4	48.0	
Unemployed	154	9.5	9.1	
Full time students	665	41.4	42.9	
**Region**				
One	385	23.8	24.2	
Two	801	49.5	50.1	
Three	431	26.7	25.7	

* The National Average data came from the 2008 census published by the National Bureau of Statistics.

Among the 124 eligible people who did not participate in the study, 41 were not at home during the visits and 83 declined the interview. Most of the people who declined to participate in the interview did so because they did not want to recall the terrible events surrounding the earthquake, while others were concerned about their privacy. Nevertheless, there were no significant differences in socio-demographic characteristics, such as age, gender, marital status, job, years of education, and shelter distributions between the respondents and non-respondents.

### Instrument

Three sets of data were collected, relating to socio-demographic characteristics, HRQoL, and social support.

#### Socio-demographic characteristics

Data about socio-demographic characteristics included age, gender, job, marital status, and level of education.

#### Quality of life

HRQoL has been defined as an "individual's perception of their position in life in the context of the culture and value systems in which they live and in relation to their goals, expectations, standards and concerns" [26]. It was measured by the Medical Outcomes Study Short Form-36 (MOS SF-36). The SF-36 contains 36 items, measuring eight domains of HRQoL: physical functioning (PF,10 items), role limitations due to physical health problems (RP, 4 items), bodily pain (BP, 2 items), general health perceptions (GH, 5 items), vitality (VT, 4 items), social functioning (SF, 2 items), role limitations due to emotional problems (RE, 3 items), and mental health (MH, 5 items). One additional item evaluates the change in health over the past year [27]. The score in each domain of the SF-36 was linearly transformed into a standard score, ranging from 0 to100 [27,28], with a higher score reflecting better perceived health. The SF-36 was chosen because it is a general instrument that can apply to a variety of populations for both clinical and public health purposes [27,29], and it has been validated in China where a general population norm is available [30-34].

#### Social support

Social support was defined as "assistance and protection given to others, especially to individuals" [35]. It was measured by the Social Support Rating Scale (SSRS). The SSRS contains 10 items, measuring three dimensions of social support: subjective support (4 items), objective support (3 items), and support-seeking behavior (3 items). Subjective support reflects the perceived interpersonal network that an individual can count on. Objective support reflects the degree of actual support an individual received in the past. Support-seeking behavior refers to the pattern of behavior that an individual utilizes when seeking social support. Item scores of the SSRS were simply added up, generating a total support score ranging from 12 to 66, a subjective support score ranging from 8 to 32, an objective support score ranging from 1 to 22, and a support-seeking behavior score ranging from 3 to 12, respectively. Higher scores indicate stronger social support. Cultural adaptation of the SSRS has been undertaken in China. The two-month test-retest reliability of the SSRS exceeded 0.92 [36]. The SSRS has been applied in a wide range of Chinese populations because of its high reliability and validity [37-39].

### Procedure and Data analysis

The 12 interviewers came from the staff of the Department of Social Medicine at Sichuan University. A seven-day training workshop, which included field tests of the instruments, was undertaken. The first 152 completed questionnaires were re-administered by a different interviewer. The inter-rater reliabilities (К) ranged from 0.85 to 0.90.

Written informed consent was obtained before the questionnaire survey. Each participant was interviewed by a trained interviewer in a private place at the shelter. The questionnaire took 30 minutes to complete.

The average values and standard deviations of HRQoL in the study population were calculated and compared with the age-adjusted norms of the general population using student t test. The data for the general population came from a questionnaire survey in a stratified randomly selected sample in the study region undertaken in 1999 (the procedure adopted in the 1999 survey was also used in this study). A total of 2249 respondents completed the survey. Details of the survey and results have been reported elsewhere [34].

Two strategies were employed to determine the association between HRQoL and social support. Firstly, the SF-36 scores were dichotomized according to whether they fell below versus at or above the age- and gender-adjusted norms [40], and then multivariate logistic regression analysis was performed with HRQoL as the dependent variable, social support as an independent variable, and socio-demographic characteristics as confounding variables.

Secondly, a canonical correlation analysis (CCA) was conducted using the three social support variables (x) as predictors of the eight quality of life variables (y) to further examine the relationships between the multiple dimensions of HRQoL and social support. CCA is a multivariate analysis technique. It allows simultaneous comparison between two groups of variables through examining the correlation between two synthetic variables. The observed variables in each group have to be combined into one synthetic variable, which is referred to as a canonical variable. CCA can minimize the probability of committing a type I error where researchers claim a significant result when it is not. It avoids the increased risk of type I error introduced by repeated statistical tests performed on one variable in a data set. CCA also has the advantage of being able to examine multiple causes and multiple effects between complex constructs, and to identify a variable's importance in terms of degree and directionality, which might otherwise be missed by univariate methods [41]. The observed variables with a significant impact on the canonical variables were judged by the canonical loadings. An absolute value greater than 0.3 was often used for identifying meaningful loadings [42].

All analyses were carried out using the Statistical Package for Social Sciences (SPSS) 15.0. A statistical significance was deemed present when the p-value was less than 0.05.

## Results

### HRQoL of respondents

The earthquake survivors had significantly lower scores in all of the eight domains of the SF-36 than the age-adjusted norms of general population in the region (Table 2). However, the gaps in mental health domains (i.e. VT, SF, RE and MH) were larger than those in physical health domains (i.e. PF and BP). Similar results were found when the scores of the respondents were compared with job-adjusted norms of the general population in the region.

**Table 2:** HRQoL scores of respondents in the survey of survivors eight months after the 2008 Wenchuan earthquake measured by SF-36, Mean (Standard Deviation)

SF-36 Domains	Respondents (n = 1617)	Adjusted Norms of General Population (n = 2249)*	*% Decrease*	*p *value (Student *t *test)
PF	87.75(16.92)	91.87(15.07)	4.48	< 0.001
RP	68.89(37.74)	82.97(34.70)	16.97	< 0.001
GH	55.76(17.56)	67.58(21.97)	17.49	< 0.001
BP	80.04(18.70)	83.57(21.25)	10.21	< 0.001
VT	57.57(14.59)	71.62(15.81)	19.62	< 0.001
SF	72.36(17.80)	84.96(18.06)	14.83	< 0.001
RE	62.71(39.45)	75.34(38.47)	16.76	< 0.001
MH	60.03(14.13)	72.92(15.68)	17.68	< 0.001

Note: * The "Adjusted Norms of General Population" derive from a stratified random sampling survey in the region where the earthquake struck, adjusted by the age structure of the respondents [34]

PF - Physical Functioning; RP - Role Limitations Due to Physical Health Problems; GH - General Health Perceptions; BP - Bodily Pain; VT - Vitality; SF - Social Functioning; RE - Role Limitations Due to Emotional Problems; MH - Mental Health.

Age, gender, marital status, education level, occupation and residential zones were associated with systematic variability in HRQoL. Older people were more likely to have lower-than-normal scores in performance of daily roles due to physical (RP) and emotional (RE) problems. Women were more likely to report worse physical functioning (PF) and greater role limitations due to emotional problems (RE). Single respondents were less likely to experience role limitations due to physical problems (RP) and bodily pain (BP) than married respondents. The respondents with tertiary education were more likely to perform daily roles (RP and RE) better than those with 0-6 years of education. The unemployed and full time students were more likely to report better general health than the employed. The survivors living in Zone Two and Zone Three were more likely to be adversely affected in the performance of daily roles (RP and RE) than those living in Zone One. However, the respondents living in Zone Three reported better general health (GH) and mental health (MH). The respondents living in Zone Two and Zone Three were less likely to experience bodily pain (BP) than those living in Zone One (detailed data are included in Table 3).

**Table 3:** Odds ratios (95% confidence intervals) for scoring at or above age- and sex-adjusted norms on SF-36 by Social Support in survivors eight months after the 2008 Wenchuan earthquake

*Independent Variables*	Dependent Variables, OR (95%CI)							
	
	PF	RP	GH	BP	VT	SF	RE	MH
Subjective support	*NS*	1.03* (1.01-1.05)	1.09***(1.05-1.13)	1.04**(1.01-1.07)	1.05**(1.02-1.09)	*NS*	*NS*	*NS*
Objective support	1.01***(1.07-1.17)	1.18***(1.12-1.23)	*NS*	1.07**(1.02-1.12)	1.11*** (1.05-1.18)	1.14***(1.06-1.21)	1.11***(1.06-1.16)	1.15***(1.09-1.22)
Support seeking	*NS*	1.08* (1.02-1.15)	*NS*	1.07*(1.01-1.13)	*NS*	1.12** (1.03-1.23)	1.10** (1.04-1.16)	*NS*
Age (with 16-39 as control)								
40-59	*NS*	*NS*	*NS*	*NS*	*NS*	*NS*	*NS*	*NS*
≥60	*NS*	0.31***(0.18-0.53)	*NS*	*NS*	*NS*	*NS*	0.53* (0.32-0.88)	*NS*
Gender (with men as control)								
Women	0.63***(0.52-0.78)	*NS*	*NS*	*NS*	*NS*	*NS*	0.76**(0.62-0.94)	*NS*
Marital status (with married as control)								
Single	*NS*	1.70** (1.17-2.46)	*NS*	1.58*(1.09-2.30)	*NS*	*NS*	*NS*	*NS*
Widowed/divorced	*NS*	*NS*	*NS*	*NS*	*NS*	*NS*	*NS*	*NS*
Years of education (with 0-6 years as control)								
7 ~ 9	*NS*	*NS*	*NS*	*NS*	*NS*	*NS*	*NS*	*NS*
10 ~ 12	*NS*	*NS*	*NS*	*NS*	*NS*	*NS*	*NS*	*NS*
Tertiary	*NS*	1.97***(1.36-2.86)	*NS*	*NS*	*NS*	*NS*	1.69** (1.17-2.44)	*NS*
Job (with employed as control)								
Unemployed	*NS*	*NS*	1.72* (1.03-2.87)	*NS*	*NS*	*NS*	*NS*	*NS*
Full time students	*NS*	*NS*	1.78* (1.13-2.81)	*NS*	*NS*	*NS*	*NS*	*NS*
Zone (with zone one as control)								
Zone two	*NS*	0.59***(0.45-0.78)	*NS*	1.40*(1.06-1.86)	*NS*	*NS*	0.46***(0.35-0.61)	*NS*
Zone three	*NS*	0.53***(0.39-0.72)	1.83** (1.24-2.71)	1.66** (1.21-2.26)	*NS*	*NS*	0.53***(0.32-0.88)	1.56*(1.08-2.25)

Adjusted R^2^	0.068	0.156	0.184	0.071	0.064	0.058	0.116	0.087

*p < 0.05; **p < 0.01; ***p < 0.001; *NS*: not significant.

### Social support reported by respondents

The respondents reported an average score of 37.58 (standard deviation = 7.01), 22.37 (standard deviation = 4.80), 7.82 (standard deviation = 2.59), and 7.38 (standard deviation = 1.85) for total support, subjective support, objective support and support seeking behaviors, respectively.

The level of social support varied with the socio-demographic characteristics. Those aged between 40 and 59 years reported the highest level of social support. Men had higher scores in subjective, objective and total social support than women, but lower levels of support-seeking behavior. The respondents who were married had higher scores in social support than those who were single or widowed. The respondents with fewer years of education reported higher levels of subjective, objective and total social support, but lower levels of support-seeking behavior. The respondents who were currently employed had higher levels of subjective, objective and total social support than those who were unemployed and those who were full-time students (Table 4).

**Table 4:** Social support scores of respondents in the survey of survivors eight months after the 2008 Wenchuan earthquake by socio-demographic characteristics, Mean (Standard Deviation)

	Subjective support	Objective support	Support seeking	Total social support
Age				
16-39	22.13 (4.71) *	7.57 (2.52)	7.50 (1.82)	37.19 (6.94)
40-59	23.46 (4.83) **	8.48 (2.70) **	7.15 (1.87) **	39.10 (7.03) **
≥60	20.94 (4.89) **	8.02 (2.41)	7.08 (1.97)	36.04 (6.84) **
Sex				
Male	22.68 (5.00)	8.00 (2.73)	7.31(1.90)	37.98(7.34)
Female	22.02 (4.53) **	7.62 (2.40) **	7.48(1.78) **	37.16(6.58) **
Marital status				
Married	23.70 (4.95) **	8.64 (2.75) **	7.30(1.92)	39.63 (7.31) **
Single	21.19 (4.27) **	7.06 (2.14) **	7.49(1.78)	35.75 (6.11) **
Others	20.77 (4.95)	7.58 (2.78)	7.00(1.86)	35.35 (7.00)
Years of education				
0 ~ 9	22.82 (4.87)	8.07 (2.51) **	7.20 (1.85) **	38.09 (6.95)
10 ~ 12	21.59 (4.65) **	7.79 (2.58)	7.38 (1.78)	36.75 (6.76) *
Tertiary	22.41 (4.73) *	7.46 (2.69)	7.68 (1.88) *	37.56 (7.27)
Job				
Employed	23.75 (4.68) **	8.55 (2.78) **	7.31 (1.87)	39.61 (7.03) **
Unemployed	21.28 (5.49) **	7.62 (2.50) **	7.29 (1.97)	36.19 (7.64) **
Full time students	20.97 (4.26)	6.99 (2.07) *	7.50 (1.79)	35.46 (6.08)

Total	22.37 (4.80)	7.82 (2.59)	7.38 (1.85)	37.58 (7.01)

The results of students *t *tests (for comparison between two groups) or ANOVA (for comparison between more than two groups, Bofferoni method was used for between-groups comparisons) are marked as * p < 0.05, **p < 0.01. The comparison results between the first and the second rows in each group are marked on the second row. The comparison results between the second and the third rows are marked on the third row. The comparison results between the first and the third rows are marked on the first row.

### Health related quality of life and social support

The respondents with stronger social support (measured by total social support) were more likely to report better HRQoL in all of the domains of SF-36. To control the confounding effect of the socio-demographic variables, multivariate logistic regression analysis was conducted with the SF-36 domains as dependent variables and social support and socio-demographic variables as independent variables. Despite a significant effect of age, gender, marital status, levels of education, job status, and geographic zones on HRQoL, all of the three dimensions of social support entered into the regression models with an OR greater than 1.0 for RP and BP (Table 3). Greater subjective social support was associated with better HRQoL in the domains of RP, GH, BP, and VT. Greater objective support was associated with better HRQoL in all of the domains except for GH. Greater support-seeking behavior was associated with better HRQoL in the domains of RP, BP, SF, and RE (Table 3). Although all of the regression models were statistically significant, the predictors explained only 6-18% of the variances of the SF-36 domains. More variances of the physical health domains (i.e. PF, RP, GH, and BP) were explained by the predictors than those of the mental health domains (i.e. VT, SF, RE, and MH).

The canonical correlation analysis yielded three pairs of canonical variables, with canonical correlations of 0.278 (p < 0.001), 0.154 (p < 0.001), and 0.088 (p = 0.049) for each successive pair respectively. The first two pairs of canonical variates explained 50% and 22% of the variance of the observed social support variables, and 42% and 7% of the variance of the observed quality of life variables, respectively. Therefore, the first two canonical sets were considered noteworthy.

In the first canonical set, all of the variables had a canonical loading greater than 0.3 on their own canonical variables, indicating that they had all contributed substantially to the synthetic variables. However, only two of the observed variables of HRQoL had a standardised canonical coefficient greater than 0.3 (-0.359 for GH and -0.308 for VT, respectively). This result was due to the multicollinearity that the observed variables had with each other (Table 5). Because all of the observed variables had the same sign, the results indicate that social support was positively related to quality of life, in particular, with perceived general health and vitality. In the second canonical set, objective support, subjective support, GH and MH had a canonical loading greater than 0.3 on their own canonical variables. Those variables also tended to have larger standardised canonical coefficients. Subjective support did not always correlate consistently with objective support. The expression of the canonical variable of social support 'L = -1.024 × 1 + 0.726 × 2 + 0.177 × 3', could be interpreted as a discrepancy between objective and subjective support, which the researchers believe is a better reflection of perceived social support needs than the three dimensions alone. A stronger presence of actual support given to people who see fewer available supportive networks could be reasonably argued as an indication of needs for social support intervention. In this research population, the magnitude of the discrepancy between objective and subjective support was inversely related to GH and positively related to MH.

**Table 5:** Canonical correlation analysis of social support and quality of life among survivors eight months after the 2008 Wenchuan earthquake

*Variable*	Canonical Set 1		Canonical Set 2	
		
	*Coef*	*r_s_*	* Coef*	* r_s_*
**Objective Support (x1)**	-.396	**-.653**	-1.024	**-.696**
**Subjective Support (x2)**	-.437	**-.743**	.726	**.342**
**Support Utilization (x3)**	-.571	**-.731**	.177	.221
**PF (y1)**	-.144	**-.505**	-.134	-.152
**RP (y2)**	-.249	**-.665**	-.575	-.234
**BP (y3)**	-.084	**-.534**	.229	.030
**GH (y4)**	-.359	**-.781**	.921	**.467**
**VT (y5)**	-.308	**-.793**	.155	-.050
**SF (y6)**	.110	**-.547**	-.377	-.282
**RE (y7)**	-.083	**-.534**	.418	.063
**MH (y8)**	-.281	**-.742**	-.717	**-.395**

*Coef *= Standardized canonical coefficients; r_s _= structure coefficient (canonical loadings);

Figures in bold indicates meaningful canonical loadings.

## Discussion

### HRQoL and social support of survivors

Earthquakes can damage the health of survivors and reduce their quality of life. This research has revealed that the Wenchuan earthquake survivors have worse HRQoL than the general population in all of the eight domains of the SF-36. The differences in the mental health domains appear larger than in the physical domains.

The multivariate logistic regression models identified age (being older) and gender (being a woman) as risk factors for poorer HRQoL. The respondents with tertiary education appear better able to perform their daily roles (RP and RE) than those with 0-6 years of education. These findings are consistent with other studies [34]. Single respondents are less likely to experience bodily pain and role limitations due to physical problems than the married. Surprisingly, the unemployed and full time students are more likely to report better general health. Although the survivors living in a zone with less extensive damage tend to report better general and mental health and less bodily pain, they are more likely to report difficulties in performing daily roles. These findings have to be explained in the light of the survivors' change in behavior and expectations as a result of the earthquake. We speculate that survivors with better job security and residing in a zone with less extensive damage are less likely to lower their expectations with regard to quality of life, and thereby decrease the scores of some domains of HRQoL [16].

The social support measured in this research focused on implicit support. Despite the fact that external assistance and explicit support had been deployed extensively in the earthquake-hit region, the implicit social support was seriously damaged by the event. The respondents reported significantly weaker social support than other Chinese populations, albeit a lesser difference in subjective support [43]. However, the earthquake survivors seem to have higher levels of support-seeking behavior than flood victims in spite of more seriously diminished objective, subjective and total social support [44]. According to Feng et al., this is perhaps because earthquake is more frightening than flood [44]. The impact of differences in demographic characteristics of the study populations on the reported levels of social support could not be ruled out. This study revealed significant correlations between reported social support scores and age, gender, marital status, educational levels and job status.

### Relationship between social support and HRQoL

Social support was found to be associated with HRQoL by multivariate logistic regression and canonical correlation analysis, which is consistent with the findings of Ikeuchi et al. and Chou et al. [14,45]. This study also showed that social support can only explain a small proportion of the variance of HRQoL, which is consistent with other studies [46-48]. Mizuno et al., [17] in a study of patients with gastrointestinal tract cancer, found that HRQoL is strongly correlated with Sense of Coherence, but only modestly correlated with social support. In an event that causes great stress, social support can improve HRQoL through a stress-buffering effect [49]. Social support can alleviate both psychological and physical effects resulting from an earthquake [6].

A wide variety of approaches to social support have been explored internationally. Social support can be designed specifically for a particular purpose (explicit support), or originate from implicit social networks (implicit support) [24]. A person may appeal for material assistance or seek psychological consolation from others, whether in times of crisis or simply for fun and entertainment [35]. Different dimensions of social support may play different roles in health and HRQoL [50]. Drageset et al. conducted a study of nursing home residents and found that while opportunities for nurturing is important to social functioning, reassurance of worth affects vitality [16]. Zheng et al. argued that informational and emotional support is more important than tangible support given to patients with systemic lupus erythematosus [51]. Indeed, in one study of patients with HIV/AIDS, informational support and affective support were positively associated with HRQoL but tangible support was not [52].

The study has contributed to our understanding of the impact of disasters on survivor wellbeing by exploring the roles that subjective support and objective support play in HRQoL. It has demonstrated that objective social support (support actually received) is positively associated with every domain of HRQoL (except for GH) measured by the SF-36. Interestingly, in a study of community-dwelling elderly people, Golden and colleagues found that social engagement is associated with better quality of life, but family support is not [53]. The SSRS used in this study does not differentiate whether the social support comes from family members or others outside of the family. However, family support has been heavily weighted in the SSRS. Given the fact that the appropriateness of social support is culturally dependent [22,23], further research into the association between social support network structures and HRQoL is warranted.

Subjective support is a measurement of the perceived support available to an individual; it is a mediator variable which can influence his/her behavior [54]. This study has revealed that subjective support is positively associated with most domains of HRQoL measured by the SF-36. Instrumental (or tangible) support without proper intervention in relation to mental health symptoms, on the other hand, will not necessarily strengthen the sense of subjective support. The results of this study suggest that mental health functioning is more likely to influence support-seeking behavior than physical health functioning. The canonical correlation analysis has demonstrated that mental health is positively related to the discrepancy between objective and subjective support. Lui and colleagues also found that increased PTSD symptoms are associated with diminished perception of social support available [55]. Feng et al pointed out that subjective support is a more powerful predictor of subsequent improvement of PTSD symptoms than objective support [44]. The stigmatisation of mental illness in the Chinese cultural context has often been found to prevent people from obtaining social support [56,57], which might, in turn, contribute to the large gap in mental health between the earthquake survivors and the general population. Obviously, mental health intervention is extremely important for the earthquake survivors. A proper intervention in relation to mental health will not only bring about benefit to mental health itself, but will also exert an impact on the demand of an individual for social support intervention.

This research has demonstrated that more openness to seeking social support is related to better quality of life outcomes. This finding is consistent with another study of patients with traumatic brain injury [58]. It is noticeable that those who are male, older, and less educated are less likely to seek social support. Social support is typically classified into three categories: emotional, informational, and instrumental (tangible) [59]. While emotional support is believed to be positively related to quality of life, greater instrumental support tends to be offered to those who have problems carrying out daily roles [60,61]. Further studies are needed to identify the types of social support that the earthquake survivors need.

This study has challenged the traditional measurement of social support. Social support has typically been measured by the amount of received or perceived support that an individual receives [62,63]. These measurements are not able to identify the types of support needs that are not being met, nor the appropriateness of the support. Therefore, "social support needs" becomes an important concept for planning interventions for social support. Unfortunately, neither subjective support nor objective support alone reflects social support needs adequately, due to the lack of a standard for judging the appropriateness of the support. This is further complicated by the difficulties in interpreting the implications of subjective support and objective support. The type of social support an individual needs is context dependent and contingent on culture [64]. The relationship between social support and health outcomes is often confounded by the presence of stressors. Because more tangible support might have been offered to people with medical diseases [65], the importance and satisfaction of social support felt by the individual (instead of the amount of support that an individual receives) is usually recommended by researchers as a better predictor for quality of life [66] and for assessing the need for social support intervention [59,67,68]. Given this, the authors believe that the discrepancy between objective and subjective support might be a better indicator for assessing the need for social support intervention. This discrepancy delineates four scenarios: (1) High objective - high subjective support: a high demand for social support which might simply be a reflection of high perceived availability of social support; (2) High objective - low subjective support: a high demand for social support despite a relatively low perceived availability of social support, which could be an indication of low satisfaction with social support; (3) Low objective - high subjective support: a low demand for social support; (4) Low objective - low subjective support: a low demand for social support which might be a consequence of low perceived availability of social support.

There are several limitations in this study. This study used a cross sectional design. The HRQoL and social support of the participants before the earthquake is unknown, which makes it difficult to know the extent of the impact of the earthquake on the quality of life and social support of the survivors. A causal conclusion about the association between social support and quality of life is also impossible.

## Conclusion

Living through a disastrous earthquake contributes to mental and physical impairment among survivors. Using the SF-36 as an instrument to assess the impact of earthquake on the HRQoL of earthquake survivors, we have confirmed that the Wenchuan earthquake seriously impaired the HRQoL of the survivors. The HRQoL of survivors is also influenced by the age, gender, marital status, jobs and residential zones of the survivors. Social support also plays an important role. Higher levels of subjective support, objective support and social support seeking behavior are all associated with higher HRQoL. However, subjective support does not consistently correlate with objective support. The discrepancy between objective and subjective support might be a better indicator for assessing the need for social support intervention. A higher objective and lower subjective support indicates a greater need for social support (perhaps due to low satisfaction with objective social support available), which is associated with poorer mental health. More attention needs to be paid to increasing social support for those with poorer mental health.

## Abbreviations

HRQoL: Health Related Quality of Life; PTSD: Posttraumatic Stress Disorder; PF: Physical Functioning; RP: Role Limitations Due to Physical Health Problems; BP: Bodily Pain; GH: General Health Perceptions; VT: Vitality; SF: Social Functioning; RE: Role Limitations Due to Emotional Problems; MH: Mental Health; SSRS: Social Support Rating Scale; CCA: Canonical Correlation Analysis; SD: Standard Deviation.

## Competing interests

The authors declare that they have no competing interests.

## Authors' contributions

KX carried out the survey, performed the statistical analysis and wrote the first draft of the manuscript. LCJ participated in literature review, data analysis and writing of the manuscript. LNX participated in the design of the study and writing of the manuscript. All authors read and approved the final manuscript.

## Pre-publication history

The pre-publication history for this paper can be accessed here:

http://www.biomedcentral.com/1471-2458/10/573/prepub
